# Foot placement control impairments in persons with chronic stroke become more evident during optic-flow perturbations

**DOI:** 10.1186/s12984-026-01967-y

**Published:** 2026-04-03

**Authors:** Joost Biere, Ramon J. Boekesteijn, Tom J. W. Buurke, Jorik Nonnekes, Brenda E. Groen, Noël L. W. Keijsers

**Affiliations:** 1 Department of Research, Sint Maartenskliniek, PO Box 9011, Nijmegen, 6500 GM The Netherlands; 2 Department of Sensorimotor Neuroscience, Donders Institute for Brain, Cognition and Behaviour, Radboud University, Nijmegen, The Netherlands; 3 Department of Human Movement Sciences, University of Groningen, University Medical Centre Groningen, Groningen, The Netherlands; 4 Department of Rehabilitation, Donders Institute for Brain, Cognition and Behaviour, Radboud University Medical Centre, Nijmegen, The Netherlands; 5 Centre of Expertise for Parkinson and Movement Disorders, Radboud University Medical Centre, Nijmegen, The Netherlands; 6 Department of Rehabilitation, Sint Maartenskliniek, Nijmegen, The Netherlands

**Keywords:** Stroke, Gait, Dynamic balance, Foot placement control, Reactive balance, Optic-flow, Virtual reality

## Abstract

**Background:**

Mediolateral balance during gait relies on regulation of the body’s centre of mass (CoM) through accurate foot placement. Despite weaker CoM-foot placement coupling in persons with chronic stroke (PwCS), they often maintain dynamic balance under steady-state conditions. In daily life, unexpected perturbations often require greater reliance on reactive balance control, which may be particularly challenging for PwCS but remains underexplored. This study examined the effect of destabilizing optic-flow perturbations on step-to-step foot placement control in PwCS compared with controls, relative to steady-state gait.

**Methods:**

Twenty PwCS and 16 controls walked on an instrumented treadmill in a virtual reality environment under three conditions: unperturbed gait and continuous mediolateral optic-flow perturbations of moderate and strong intensity. Perturbation effects on CoM dynamics were quantified using the excursion and variability of the extrapolated CoM (xCoM). Step-to-step foot placement control was assessed as the RMSE between actual and model-predicted foot placements from a linear regression using CoM position and velocity. Mean and variability of the margin of stability were also calculated. Group (paretic, non-paretic, control), condition, and their interaction were evaluated using linear mixed effects models, and exploratory correlations of foot placement deviation across conditions were examined.

**Results:**

Optic-flow perturbations increased xCoM variability (*p* < 0.001), with no group × condition interaction (*p* ≥ 0.08). Foot placement deviation (RMSE) was higher in PwCS across all conditions (all *p* < 0.01) and, compared to controls, showed a significant group × condition interaction at strong intensity (paretic and non-paretic both *p* = 0.01), but not at moderate intensity (both *p* = 0.07). Foot placement control did not differ between paretic and non-paretic legs (*p* ≥ 0.60). PwCS showed larger average margins of stability and greater increases in variability with perturbation intensity than controls (all *p* ≤ 0.01). Correlations between unperturbed and perturbed conditions were weak to moderate (*r* = 0.2–0.45) but strong between perturbation intensities (*r* ≥ 0.68).

**Conclusions:**

PwCS exhibited impaired mediolateral foot placement control, which became more pronounced when reactive balance demands increased. Challenging balance through continuous optic-flow perturbations exacerbates impairments in step-to-step balance control after stroke.

**Supplementary Information:**

The online version contains supplementary material available at 10.1186/s12984-026-01967-y.

## Introduction

Persons with chronic stroke (PwCS) often exhibit sensorimotor impairments, including muscle weakness [1, 2], impaired selective motor control [1], spasticity [3, 4], and proprioceptive deficits [5]. These impairments often lead to compromised balance during gait, increasing fall risk [6], limiting daily activities and social participation [7], and ultimately reducing quality of life [8].

Maintaining balance during gait requires continuous control of the body’s centre of mass (CoM) relative to a shifting base of support (BoS), which is particularly challenging in the mediolateral (ML) direction [9–11]. In healthy adults, ML balance is primarily maintained through foot placement control [12]. Previous work has shown that the location of ML foot placement is strongly related to CoM dynamics, with CoM position and velocity explaining over 80% of foot placement variance during unperturbed, steady-state gait [13]. Accurate foot placement control requires both reliable sensory information and adequate motor control [12, 14]. Stroke related impairments in proprioception and motor control [15–17] disrupt this mechanism. Consequently, PwCS show greater step-to-step variability in foot placement location and a weaker coupling between CoM dynamics and foot placement, particularly on the paretic side [18–20].

Despite these alterations, many PwCS are able to maintain dynamic balance during steady-state walking in predictable environments, partly due to proactive, compensatory wider stepping [21]. In daily life, however, balance during gait is often challenged by internal or external perturbations that require effective reactive balance control [6, 22]. Assessing foot placement control under destabilizing conditions is therefore particularly relevant as these conditions impose greater challenges on the impaired sensorimotor control of PwCS and may amplify foot placement control impairments relative to steady-state gait, yet remains relatively underexplored in PwCS.

Optic-flow perturbations offer a non-invasive method to challenge balance control. These perturbations manipulate visual information about self-motion that can bias state estimation and sensory integration and are interpreted by the central nervous system as instability, thereby eliciting reactive balance responses [23–25]. In contrast to mechanical perturbations such as slips or trips, optic-flow perturbations do not impose external forces on the body or base of support, avoiding confounding mechanical effects that complicate interpretation of the resulting gait mechanics. Although the destabilizing effect of visual-input manipulations on gait varies between individuals [26–28], optic-flow perturbations consistently alter CoM dynamics during walking and can be applied continuously across many strides [23, 24, 29–31]. By destabilizing gait and amplifying variability in CoM dynamics, these optic-flow perturbations increase the need for accurate step-to-step foot placement control, potentially revealing impairments that are less apparent, or can be compensated for during unperturbed, steady-state gait.

This study examined mediolateral foot placement control in PwCS and controls during treadmill walking under three conditions: unperturbed steady-state gait and continuous mediolateral optic-flow perturbations of moderate and strong intensity. The primary aim was to determine whether optic-flow perturbations amplify stroke-related impairments in step-to-step foot placement control that may be less apparent during unperturbed walking. To address this aim, we formulated three hypotheses (H1–H3), with H3 serving as the primary hypothesis. We expected (H3) a condition-by-group interaction such that PwCS, particularly on the paretic side, would show increasingly larger errors in step-to-step foot placement control as perturbation intensity increased, compared with controls. To support interpretation of this primary hypothesis, we included two additional hypotheses. H1 served as a methodological check, predicting that optic-flow perturbations would alter mediolateral CoM dynamics in PwCS and controls, resulting in increased mediolateral extrapolated CoM (xCoM) variability with increasing perturbation intensity. After establishing perturbation effects on xCoM variability, H2 assessed overall group differences in foot placement control across conditions. We expected that PwCS would exhibit greater errors in step-to-step foot placement control during unperturbed gait, as has been reported previously [18–20], as well as during perturbed gait conditions. Finally, we included an exploratory analysis aimed to determine whether foot placement deviation during unperturbed walking is informative for an individual’s foot placement deviation during perturbed conditions.

## Methods

### Participants

Twenty PwCS and sixteen controls participated in this study. PwCS were more than 6 months after stroke onset and eligible for inclusion if they exhibited self-reported hemiparetic gait and balance impairments. PwCS were included regardless of age, whereas controls were restricted to 50–75 years. Exclusion criteria for both groups were (1) significant pain during gait, (2) (additional) orthopaedic, neurologic, respiratory, or muscular conditions that would likely affect gait, (3) uncorrected visual impairments or spatial neglect, or (4) inability to follow verbal instructions. All participants provided written informed consent prior to the measurements. The Medical Research Ethics Committee of Eastern Netherlands (file number 2024–17166) exempted this study from ethical approval, as it was not subjected to the Medical Research Involving Human Subject Act according to Dutch law.

### Experimental procedures

#### Apparatus

**Fig. 1:**
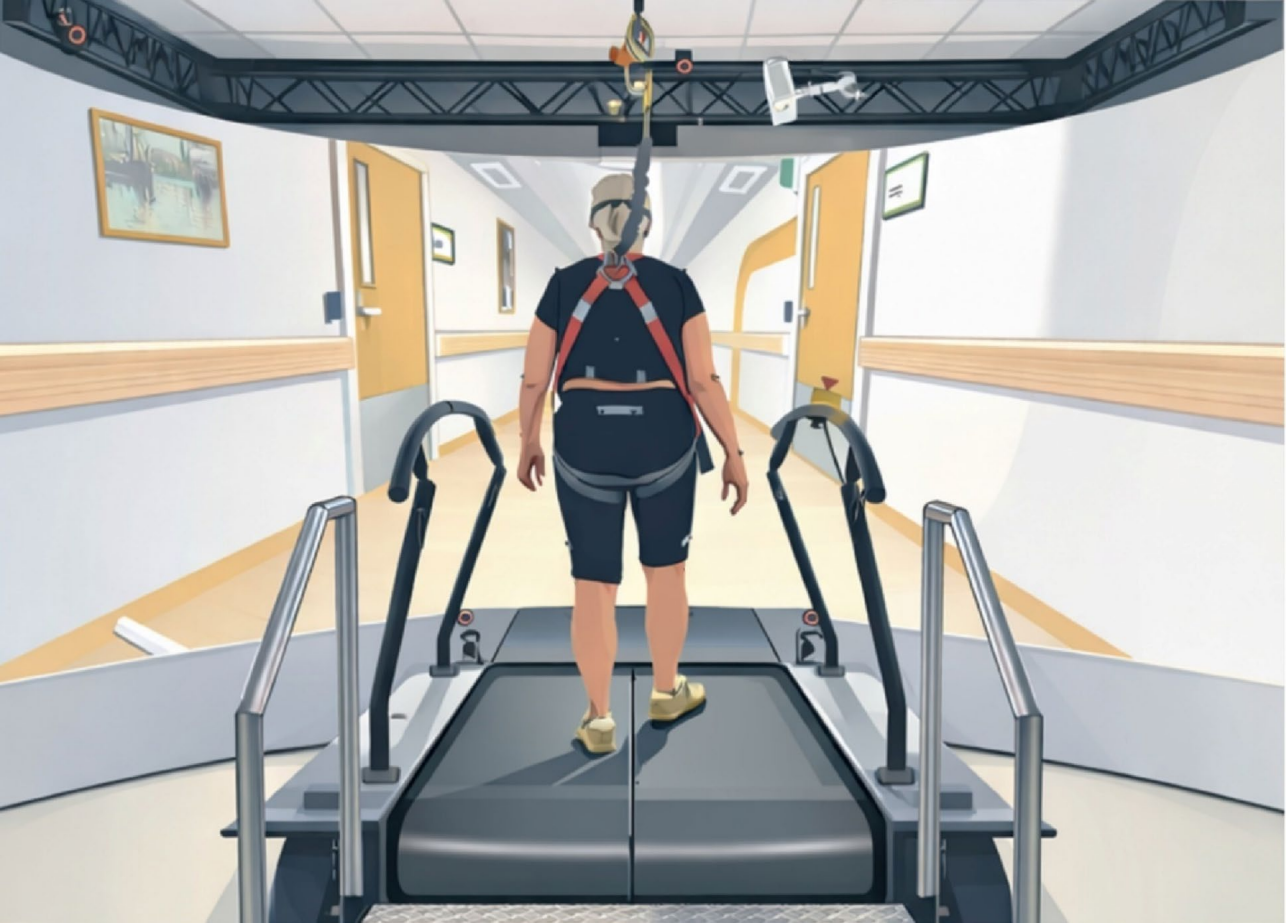
An illustration of the Gait Real-time Analysis Inter-active Lab (GRAIL) and the virtual hallway environment

Participants completed a series of walking bouts on an instrumented treadmill (GRAIL, Motek Medical BV, the Netherlands) while immersed in a virtual hallway environment. The virtual scene was projected on a semi-cylindrical screen located 2.3 m from the treadmill centre and was matched to treadmill speed to provide congruent optic-flow. Kinematic data were collected at 100 Hz using ten infrared cameras (VICON, Oxford, United Kingdom). In total, 26 passive markers were placed at the following anatomical landmarks: lateral malleoli, second metatarsal heads, calcanei, lateral shanks, lateral femoral epicondyles, lateral thighs, posterior superior iliac spines, anterior superior iliac spines, ulnar styloid processes, lateral humeral epicondyles, acromion processes, xiphoid process, suprasternal notch, and 7th and 10th thoracic vertebrae. A non-weight bearing safety harness suspended from the ceiling ensured participant safety (Fig. 1).

#### Experimental protocol

First, comfortable walking speed was determined using a standardized protocol [32] in which treadmill speed was gradually increased until participants reported a comfortable pace, followed by a gradual decrease from a faster speed until a comfortable pace was again reported. The average of the two reported speeds was used as the participant’s comfortable walking speed. Prior to data collection, participants completed a familiarization period of at least four minutes of steady-state walking at their comfortable walking speed in the virtual hallway environment. Subsequently, they performed the following three conditions in randomized order: unperturbed, steady-state gait (3 min and 20 s), moderate perturbation (4 min and 20 s), and strong perturbation (4 min and 20 s). PwCS completed all trials at 90% of comfortable walking speed to account for the typically observed slowing of gait when facing destabilizing conditions [33], and controls at 50% of their comfortable speed to eliminate the confounding effect of gait speed on foot placement control [32], allowing for group-comparisons at a similar speed. To eliminate stationary cues from treadmill handrails and other static elements, all participants wore dribble goggles that blocked the lower part of the visual field. After each trial, participants rated their fatigue using the Borg Rating of Perceived Exertion scale [34] and were asked to rest when their fatigue rated above a Borg-score of 13. Furthermore, participants were asked to rate the perceived balance difficulty for each condition using a 0–10 scale in response to the question: “*How difficult was it to maintain your balance during this walking bout?*”.

#### Optic-flow perturbation characteristics

Continuous mediolateral optic-flow perturbations were applied by rotating the virtual hallway around a stationary vanishing point at the end of the hallway. These perturbations consisted of a sum of three zero-phase sine waves at 0.125 Hz, 0.250 Hz, and 0.442 Hz, with the 0.125 Hz and 0.442 Hz frequencies being half the amplitude (A) of the 0.250 Hz frequency (Eq. 1).

1 $$\:\:\:\begin{array}{l}Perturbation\:\left(t\right)=A\mathrm{sin}\left(0.25*2\pi\:t\right)+0.5A\mathrm{sin}\left(0.125*2\pi\:t\right) \\ \hfill +0.5A\:\mathrm{s}\mathrm{i}\mathrm{n}\left(0.442*2\pi\:t\right)\end{array}$$

The two perturbation conditions either had a strong intensity (A = 0.350), and a moderate intensity (A = 0.175). These optic-flow perturbation characteristics have been used in previous work [29, 35, 36], and induce lateral balance corrections rather than heading corrections.

### Data analysis

#### Data processing

Force and kinematic data were processed and analysed in MATLAB (R2023b, MathWorks Inc., Natick, MA, USA). The first fifteen and last five seconds of each trial were excluded to ensure steady-state gait by accounting for the start and stop phase, respectively. The first minute of the remaining data was excluded to ensure a continuous and consistent perturbation level across conditions, as this period is characterized by heterogeneous balance responses and strong visuomotor adaptation [29, 36]. This resulted in two minutes of data in the regular gait condition and three minutes in the perturbation conditions. Marker trajectories were low-pass filtered using a second-order, zero-phase Butterworth filter with a 20 Hz cut-off frequency. The whole-body centre of mass (CoM) was estimated as a weighted sum of nine segmental CoM estimates [37]. Gait events were based on foot markers. Initial contact was the instant the calcaneus marker started moving opposite to the walking direction, toe-off was when the second metatarsal marker started moving in the walking direction, and midstance was the midpoint of the anteroposterior distance between initial contact and toe-off [38]. To control for potential confounding effects of sporadic handrail use, handrail contacts were determined through visual inspection of the trial recordings. All strides with handrail contact, as well as the subsequent stride, were excluded from the analyses.

#### Outcome measures

The effect of optic-flow perturbations on mediolateral CoM dynamics (H1) was evaluated using the variability of the xCoM [39] excursion, hereafter referred to as xCoM variability. xCoM variability was quantified as the standard deviation over the mediolateral distances between mediolateral xCoM peaks (xCoM excursion) within strides, thereby capturing step-to-step fluctuations in CoM dynamics.

To evaluate foot placement control during perturbed and unperturbed walking in both groups (H2), the primary outcome, *foot placement deviation*, was derived from a linear regression model describing the relationship between CoM dynamics and mediolateral foot placement [13]:

2 $$\:{FP}_{act\:}=\:{\beta\:}_{pos\:\:}\cdot\:{{COM}_{pos\:}}_{\:}+{\beta\:}_{vel\:\:}\cdot\:{{COM}_{vel\:}}_{\:}+\epsilon\:,\:$$

in which foot placements are fitted using a linear regression based on CoM position ($\:{COM}_{pos\:}$) and velocity ($\:{COM}_{vel\:}$) at initial contact, with their corresponding regression coefficients $\:{\beta\:}_{pos\:}$and $\:{\beta\:}_{vel}$​ and the model error $\:\epsilon\:$. Actual foot placements ($\:{FP}_{act\:}$) were defined as the demeaned mediolateral distance of the swing foot relative to the contralateral stance foot at midstance. 

*Foot placement deviation* was defined as the root mean square error (RMSE) between fitted and actual foot placements, and aims to capture the accuracy of step-to-step foot placement control by quantifying the level of error, or deviation, from the fitted foot placements. Lower RMSE values indicate less step-to-step error between the observed foot placements and those predicted from the CoM state, reflecting more precise foot placement control. As a control measure, *foot placement adherence* (the model’s coefficient of determination; R²) quantified the proportion of foot placement variance explained by CoM dynamics, and aimed to capture the overall relationship between foot placements and CoM dynamics. Only trials with an R² above 0.5 were included in the primary analysis, implying that at least 50% of lateral foot placement variance should be explained by CoM position and velocity. This threshold was set to allow for a meaningful interpretation of foot placement deviation and ensuring an adequate model fit [40].

Secondary outcome measures included the model regression coefficients $\:{\beta\:}_{pos\:}$and $\:{\beta\:}_{vel}\:$, mediolateral margin of stability (MoS), MoS variability, the average xCoM excursion, and the perceived task difficulty (0–10 scale). The margin of stability (MoS) was defined as the minimum mediolateral distance between the xCoM and the midpoint of the line connecting the calcaneus and second metatarsal markers during single stance. MoS variability was the standard deviation of MoS across steps. Additionally, the following spatiotemporal gait parameters were extracted and presented in Supplemental Table S1: step length, step width, step width variability, cadence, stance phase duration, swing phase duration, and double support phase duration. Steps were categorized as paretic or non-paretic for PwCS, and only left steps were analyzed for control participants.

#### Statistical analysis

Differences between groups and experimental conditions were assessed using linear mixed-effects models (LMM) with a random effect for participant (intercept), for primary and secondary outcome measures. Fixed effects included ‘group’ (PwCS and controls) and ‘condition’ (unperturbed, moderate perturbation intensity and strong perturbation intensity), and their interaction (Eq. 3).

3 $${\mathrm{Outcome}}={\mathrm{Group}}*{\mathrm{Condition}}+\left( {{\mathrm{1}}\left| {{\mathrm{Participant}}} \right.} \right)$$

For outcome measures in which the paretic and non-paretic leg were analyzed separately, ‘group’ was replaced with ‘leg’, which is a three-level factor (paretic, non-paretic, and control). For these outcomes, an additional random effect for leg within participant was added to account for the dependence between paretic and non-paretic legs (Eq. 4).

4 $${\mathrm{Outcome}}={\text{Leg }}*{\text{ Condition}}+\left( {\left. 1 \right|{\mathrm{Participant}}} \right)+\left( {\left. 1 \right|{\mathrm{Participant}}:{\mathrm{Leg}}} \right)$$

For both equations, the control group and the unperturbed condition were set as reference levels. Model assumptions of normality of residuals and homogeneity of variance were evaluated through visual inspection of Q-Q plots and residuals versus fitted plots. When significant main effects or interactions were found, post-hoc comparisons of estimated marginal means (EMMs) with Tukey adjustment were used to compare conditions within groups and groups within conditions.

Furthermore, we included an exploratory analysis aimed to determine whether foot placement deviation during unperturbed walking is informative for an individual’s foot placement deviation during perturbed conditions. To this end, correlations of foot placement deviation values between conditions were computed within each group. Correlation coefficients were Pearson’s r or Spearman’s ρ, depending on normality of the data distribution. Correlation coefficients were considered small (< 0.30), moderate (0.30 to 0.50), or large (≥ 0.50) [41, 42].

The number of participants needed was determined a priori by a power analysis (paired, two-sided, α = 0.05, power = 0.80) for the primary within-subject change in foot placement deviation (primary outcome measure). Based on pilot data in PwCS (*n* = 3), we assumed an expected change of 0.8 cm and an SD of paired differences of 0.8 cm (Cohen’s d = 1.0), indicating that at least 15 PwCS were required. To account for 10% missing data, we aimed to include at least 17 PwCS who were able to complete at least one perturbed condition.

Participant characteristics and spatiotemporal gait parameters were presented descriptively as mean ± standard deviation for normally distributed data, as median and interquartile range for non-normally distributed data, or as ratios for categorical variables.

LMM’s were fitted in R (version 4.4.0) using the ‘lmerTest’ package. Statistical significance was determined at *p* < 0.05.

## Results

### Participants

Participant characteristics are shown in Table 1. Of the 20 PwCS and 16 controls enrolled, three PwCS could not perform the perturbation trials without handrail support and were excluded from the analysis, leaving 17 PwCS and 16 healthy controls (HC). Two of the 17 analyzed PwCS completed only the unperturbed and moderate perturbation conditions. For one of the 17 analyzed PwCS, the paretic leg was excluded from analysis in all conditions as foot placement adherence was below 0.5.

**Table 1:** Participant characteristics

	PwCS	Controls	*p*-value
*N*	17	16	
Age (years)	66 ± 8	65 ± 8	0.85
Sex (male/female)	14/3	11/5	0.36
Weight (kg)	89 ± 13	81 ± 17	0.14
Height (cm)	178 ± 7	177 ± 9	0.96
Comfortable walking speed (m/s)	0.78 ± 0.15	1.26 ± 0.16	**< 0.001**
Walking speed during experimental conditions (m/s)	0.69 ± 0.13	0.63 ± 0.08	0.09
MiniBEST score (0–28)	18 ± 2	26 ± 2	**< 0.001**
Activities-Specific Balance Confidence scale (0–100%)	74 ± 16	96 ± 6	**< 0.001**
Affected leg (left/right)	7/10		
Time since stroke (months)	45 [11–88]		

Values are presented as mean ± SD or median [IQR]. Between-group comparisons are independent t-tests if normally distributed or Mann-Whitney U tests if non-normally distributed. Male – female ratios between groups were tested with a Chi-square test. Bold p-values indicate a statistically significant difference between persons with chronic stroke (PwCS) and controls

### Linear mixed-effects models

Model outputs for the fixed effects and interactions of the primary and secondary outcome measures are presented in Table 2, and post-hoc estimated marginal means (EMMs) are summarized in Table 3. Inspection of Q–Q plots and residuals-versus-fitted value plots indicated no substantial violations of normality or homoscedasticity assumptions. Descriptive statistics of spatiotemporal gait parameters per group and condition are presented in Supplemental Table S1.

### Perturbation effect on CoM dynamics (H1)

**Fig. 2:**
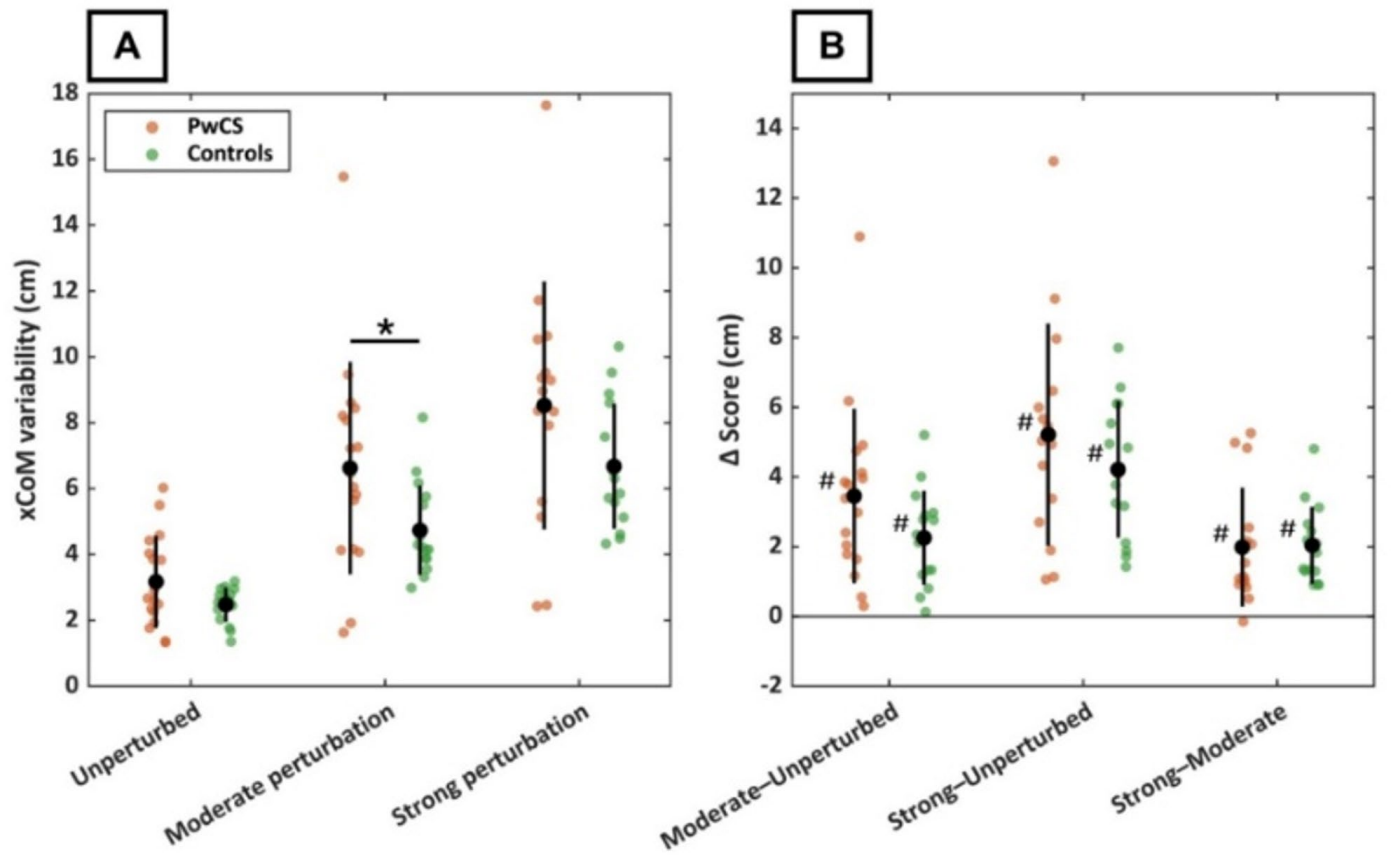
Extrapolated centre of mass (xCoM) excursion variability in persons with chronic stroke (PwCS; orange) and controls (green) across three optic-flow conditions: unperturbed, moderate perturbation intensity, and strong perturbation intensity. Panel A shows the absolute xCoM excursion variability for each condition, with single data points representing individual participants. Panel B shows within-participant changes in xCoM variability between conditions (∆ scores). In both panels, black markers indicate group means, and black bars represent group standard deviations. * = statistically significant group difference (*p* < 0.05); # = statistically significant increase between conditions (*p* < 0.05)

Figure 2A shows xCoM variability for both groups and all conditions, with corresponding between-condition differences in Fig. 2B. Regarding H1, the LMM revealed no significant group × condition interaction (moderate, *p* = 0.08; strong, *p* = 0.34) and no group effect (*p* = 0.40), indicating a similar condition effect and no baseline difference between groups. There was a strong positive condition effect at both the moderate (*p* < 0.001) and strong (*p* < 0.001) perturbation intensities, reflecting increased xCoM variability under perturbed conditions. Post-hoc EMM contrasts showed that, compared to unperturbed gait, xCoM variability increased by 2.3 cm (95% CI [0.93, 3.6]) during moderate and by 4.2 cm (95% CI [2.9, 5.6]) during strong perturbation intensity. In addition, xCoM variability increased by 2.0 cm (95% CI [0.62, 3.3], *p* = 0.004) from moderate to strong perturbation intensity.

Furthermore, secondary outcome measures showed no significant group × condition interactions for average xCoM excursion (*p* = 0.66–0.81) or perceived task difficulty (*p* = 0.40–0.57), indicating similar condition effect in both groups. A significant group effect indicated that PwCS exhibited larger average xCoM excursions (*p* ≤ 0.03) and higher perceived task difficulty (*p* ≤ 0.003) compared to controls. In addition, a strong condition effect was found for both measures, with values increasing from unperturbed walking to moderate and strong perturbation intensities (all *p* < 0.001), and further from moderate to strong intensity (all *p* < 0.001).

### Foot placement control (H2–H3)

**Fig. 3:**
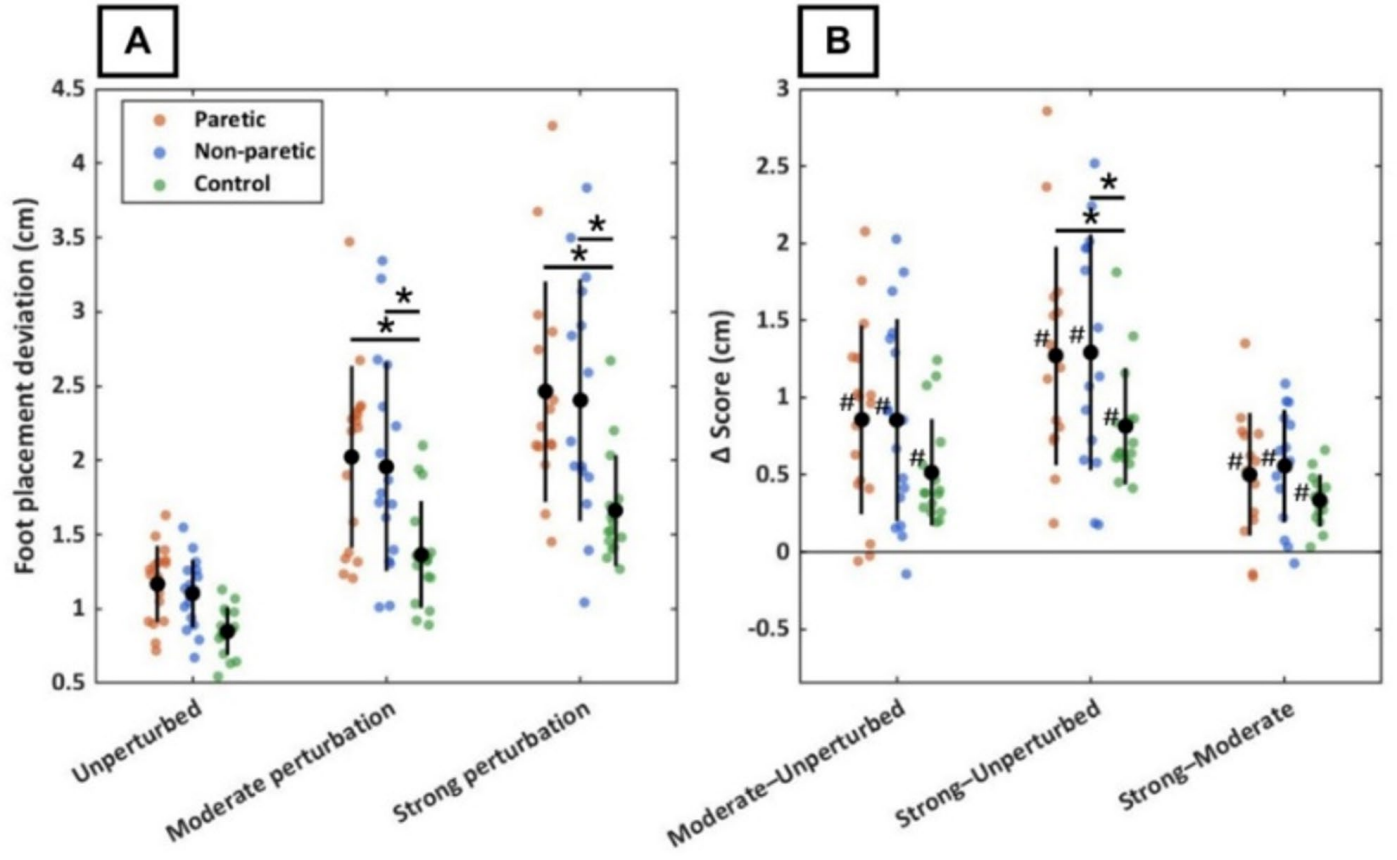
Foot placement deviation in persons with chronic stroke (paretic leg: orange; non-paretic leg: blue) and controls (green) during three optic-flow conditions: unperturbed, moderate perturbation intensity, and strong perturbation intensity. Panel A shows the foot placement deviation for each condition, with single data points representing individual participants. Panel B shows within-participant differences in foot placement deviation between conditions (∆ scores). In both panels, black markers indicate group means, and black bars represent group standard deviations. * = statistically significant group difference (*p* < 0.05); # = statistically significant increase between conditions (*p* < 0.05)

Figure 3A shows foot placement deviation in all legs (paretic, non-paretic, and control) and conditions, with the corresponding between-condition differences in Fig. 3B. Regarding H3, the LMM revealed significant leg × condition interactions for foot placement deviation at the strong perturbation intensity (Table 2), with both the paretic (*p* = 0.01) and non-paretic legs (*p* = 0.01) showing an increase in foot placement deviation relative to controls. Interaction effects for the moderate perturbation intensity did not reach statistical significance for either leg (paretic: *p* = 0.07; non-paretic: *p* = 0.07). The non-significant interaction effect for the moderate perturbation condition allowed for the interpretation of a significant condition effect from unperturbed to moderate perturbation intensity (*p* < 0.001). Regarding H2, post-hoc EMM contrasts revealed that foot placement deviation was significantly higher in both legs of PwCS compared to controls in the perturbed conditions (*p* < 0.001), but not for the unperturbed condition (paretic: *p* = 0.07; non-paretic: *p* = 0.15).

Post-hoc EMM contrasts further showed that foot placement deviation increased for all legs with increasing perturbation intensity, as detailed below. In controls, deviation increased by 0.52 cm (95% CI [0.25, 0.79], *p* < 0.001) from unperturbed gait to moderate perturbation intensity, by 0.82 cm (95% CI [0.55, 1.1], *p* < 0.001) from unperturbed gait to strong perturbation intensity, and by 0.31 cm (95% CI [0.03, 0.58], *p* = 0.028) from moderate to strong perturbation intensity. In PwCS, foot placement deviation in the paretic leg increased by 0.86 cm (95% CI [0.60, 1.1], *p* < 0.001) from unperturbed gait to moderate perturbation intensity, by 1.32 cm (95% CI [1.1, 1.6], *p* < 0.001) from unperturbed gait to strong perturbation intensity, and by 0.46 cm (95% CI [0.20, 0.73], *p* < 0.001) from moderate to strong perturbation intensity. Corresponding increases in the non-paretic leg were 0.85 cm (95% CI [0.60, 1.1], *p* < 0.001), 1.3 cm (95% CI [1.1, 1.6], *p* < 0.001), and 0.48 cm (95% CI [0.21, 0.74], *p* < 0.001), respectively. There were no significant differences between the paretic and non-paretic leg in any condition (all *p* > 0.60).

**Fig. 4:**
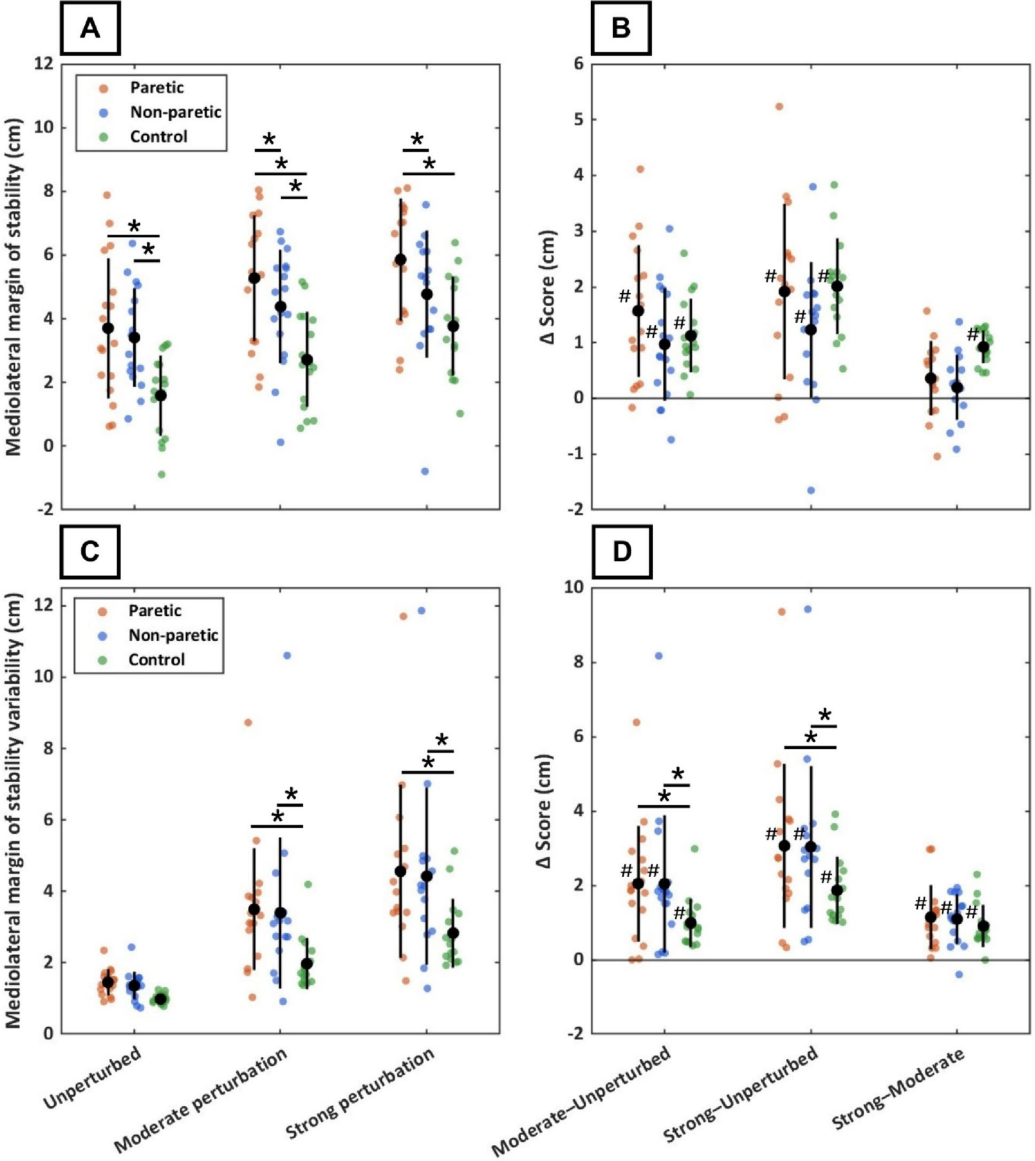
Foot placement deviation in persons with chronic stroke (paretic leg: red; non-paretic leg: blue) and controls (green) during three optic-flow conditions: unperturbed, moderate perturbation intensity, and strong perturbation intensity. Panel A shows the foot placement deviation for each condition, with single data points representing individual participants. Panel B shows within-participant differences in foot placement deviation between conditions (∆ scores). In both panels, black markers indicate group means, and black bars represent group standard deviations

Furthermore, secondary outcome measures showed no significant leg × condition interactions (*p* ≥ 0.15), and no significant condition effects (*p* ≥ 0.35) for either the CoM position or CoM velocity regression coefficient. For the CoM position coefficient, post-hoc EMM contrasts revealed no statistically significant differences between conditions or legs (*p* ≥ 0.09). For the CoM velocity coefficient, the paretic leg showed lower values in all conditions compared to both controls (*p* ≤ 0.005) and the non-paretic leg (*p* ≤ 0.03). Moreover, significant leg × condition interactions were observed for mediolateral MoS variability at both the moderate (*p* = 0.02) and strong (*p* ≤ 0.001) perturbation intensities, with larger increases for the paretic and non-paretic legs relative to controls. No significant leg × condition interaction was found for mediolateral MoS (*p* ≥ 0.08). In all conditions, PwCS demonstrated larger average MoS values than controls (*p* ≤ 0.007), and MoS was significantly larger in the paretic compared with the non-paretic leg for both perturbation intensities (both *p* ≤ 0.01). In addition, a strong condition effect was found for both MoS and MoS variability, with values increasing from unperturbed walking to moderate and strong perturbation intensities (all *p* < 0.001), and further from moderate to strong intensity (all *p* < 0.001). Figure 4 presents the average MoS and MoS variability for all legs and conditions, along with the corresponding between-condition differences.

### Foot placement control in unperturbed versus perturbed gait

Exploratory correlation analysis of foot placement deviation between conditions revealed weak to moderate relationships between unperturbed gait and moderate perturbation intensity in controls (Spearman’s ρ = 0.45, *p* = 0.09), the paretic leg (Pearson’s *r* = 0.22, *p* = 0.40), and the non-paretic leg (*r* = 0.36, *p* = 0.15). Similar patterns were observed between unperturbed gait and strong perturbation intensity for controls (*r* = 0.20, *p* = 0.47), the paretic leg (*r* = 0.31, *p* = 0.26), and the non-paretic leg (*r* = 0.37, *p* = 0.17). In contrast, foot placement deviation between moderate and strong perturbation intensities were strongly correlated in controls (ρ = 0.68, *p* = 0.007), the paretic (*r* = 0.84, *p* < 0.001) and non-paretic legs (*r* = 0.90, *p* < 0.001).

Figure 4. Mediolateral margin of stability (MoS) and MoS variability in persons with chronic stroke (paretic leg: orange; non-paretic leg: blue) and controls (green) during three optic-flow conditions: unperturbed, moderate perturbation intensity, and strong perturbation intensity. Panel A shows the average mediolateral MoS for each condition, with single data points representing individual participants. Panel B shows within-participant differences in average mediolateral MoS between conditions (∆ scores). Panel C shows mediolateral MoS variability, expressed as the standard deviation across steps, for each condition. Panel D shows within-participant differences in mediolateral MoS variability between conditions (∆ scores). In all panels, black markers indicate group means, and black bars represent group standard deviations. * = statistically significant group difference (*p* < 0.05); # = statistically significant increase between conditions (*p* < 0.05).

**Table 2:** Fixed effects estimates for group, leg, and condition effects on primary and secondary outcome measures. Note: bold values indicate statistically significant main effects (*p* < 0.05). Asterisks mark significant effects that cannot be interpreted independently due to a significant interaction term involving the same factor

Parameter	Intercept	Interactions (Group x Condition)		Fixed effect estimates		
	Group (PwCS)	PwCS × Moderate	PwCS × Strong	Condition (Moderate)	Condition (Strong)	
xCoM variability (cm)	β = 0.64, SE = 0.76, *p* = 0.40	β = 1.3, SE = 0.71, *p* = 0.08	β = 0.70, SE = 0.74, *p* = 0.34	**β = 2.3**,** SE = 0.51**, ***p*** **< 0.001**	**β = 4.2**,** SE = 0.52**, ***p*** **< 0.001**	
xCoM excursion (cm)	**β = 2.3**,** SE = 1.1**, ***p*** **= 0.03**	β=−0.25, SE = 1.0, *p* = 0.81	β=−0.45, SE = 1.0, *p* = 0.66	**β = 2.5**,** SE = 0.73**, ***p*** **< 0.001**	**β = 4.1**,** SE = 0.74**, ***p*** **< 0.001**	
Perceived task difficulty (0–10)	**β = 2.2**,** SE = 0.71**, ***p*** **= 0.003**	β=–0.33, SE = 0.58, *p* = 0.57	β=–0.49, SE = 0.59, *p* = 0.40	**β = 2.6**,** SE = 0.42**, ***p*** **< 0.001**	**β = 4.5**,** SE = 0.42**, ***p*** **< 0.001**	

Parameter	Intercepts		Interactions (Leg x Condition)					Fixed effect estimates		
	Leg (Paretic)	Leg (Non-paretic)	Paretic × Moderate		Non-paretic × Moderate	Paretic × Strong	Non-paretic × Strong	Condition (Moderate)		Condition (Strong)
Foot placement deviation (cm)	β = 0.32, SE = 0.18, *p* = 0.07	β = 0.26, SE = 0.18, *p* = 0.15	β = 0.34, SE = 0.19, *p* = 0.07		β = 0.34, SE = 0.19, *p* = 0.07	**β = 0.49**,** SE = 0.19**, ***p*** **= 0.01**	**β = 0.50**,** SE = 0.19**,* p* ***= 0.01***	**β = 0.52**,** SE = 0.13**, ***p*** **< 0.001**		β = 0.83, SE = 0.14, ***p*** < 0.001 *
CoM position coefficient (β)	β = 0.15, SE = 0.09, ***p*** = 0.11	β = 0.02, SE = 0.09, *p* = 0.79	β = –0.14, SE = 0.10, *p* = 0.15		β = –0.13, SE = 0.10, *p* = 0.18	β=–0.15, SE = 0.10, *p* = 0.15	β = –0.14, SE = 0.10, *p* = 0.16	β = 0.02, SE = 0.07, *p* = 0.80		β = –0.03, SE = 0.07, ***p*** **= 0.65**
CoM velocity coefficient (β)	**β = –0.14**,** SE = 0.05**, **p** **= 0.003**	β = –0.06, SE = 0.05, *p* = 0.23	β = –0.04, SE = 0.4, *p* = 0.42		β = –0.04, SE = 0.4, *p* = 0.41	β = 0.004, SE = 0.05, *p* = 0.93	β = 0.002, SE = 0.05, ***p*** = 0.96	β = 0.004, SE = 0.03, *p* = 0.91		β = –0.03, SE = 0.03, ***p*** = 0.35
Margin of stability (cm)	**β = 2.1, SE = 0.68**,*** p*** **= 0.0029**	**β = 1.9**,** SE = 0.68**,*** p*** **= 0.0073**	β = 0.37, SE = 0.40, *p* = 0.35		β = 0.13, SE = 0.40, *p* = 0.74	β=−0.28, SE = 0.41, *p* = 0.49	β = −0.73, SE = 0.41, *p* = 0.08	**β = 1.1**,** SE = 0.29**,*** p*** < **0.001**		**β = 2.0**,** SE = 0.29**,***p*** **< 0.001**
Margin of stability variability (cm)	β = 0.47, SE = 0.50, *p* = 0.35	β = 0.37, SE = 0.50, *p* = 0.45	**β = 1.1**,** SE = 0.46**, ***p*** **= 0.02**		**β = 1.1**,** SE = 0.46**, ***p*** **= 0.02**	**β = 1.3**,** SE = 0.47**, ***p*** **= 0.009**	**β = 1.2**,** SE = 0.47**,* p* ***= 0.01***	β = 0.99, SE = 0.33, ***p*** = 0.003*		β = 1.9, SE = 0.34, ***p*** < 0.001*

**Table 3:** Estimated marginal means for primary and secondary outcome measures per condition

Parameter	Controls (*n* = 16)			Persons with chronic stroke (*n* = 17)			
	Unperturbed	Moderate perturbation	Strong perturbation	Leg	Unperturbed	Moderate perturbation	Strong perturbation
xCoM excursion variability (cm)	2.5 [1.4, 3.6]	4.7 [3.7, 5.8]	6.7 [5.6, 7.8]		3.1 [2.1, 4.2]	6.7 [5.7, 7.8]	8.1 [7.0, 9.2]
xCoM excursion (cm)	9.9 [8.3, 11.4]	12.4 [10.8, 13.9]	14.0 [12.4, 15.5]		12.2 [10.7, 13.7]	14.5 [13.1, 16.0]	15.1 [13.5, 16.6]
Perceived task difficulty (0–10)	1 [0, 2]	4 [2, 5]	5 [4, 6]		3 [2, 4]	5 [4, 6]	7 [6, 8]
Foot placement deviation (cm)	0.85 [0.60, 1.1]	1.4 [1.1, 1.6]	1.7 [1.4, 1.9]	Paretic	1.2 [0.92, 1.4]	2.0 [1.8, 2.3]	2.5 [2.2, 2.7]
				Non-paretic	1.1 [0.9, 1.4]	2.0 [1.7, 2.2]	2.4 [2.2, 2.7]
Foot placement adherence (R^2^)	0.88 [0.83, 0.92]	0.92 [0.87, 0.96]	0.92 [0.87, 0.97]	Paretic	0.83 [0.78, 0.87]	0.86 [0.81, 0.90]	0.85 [0.81, 0.90]
				Non-paretic	0.87 [0.83, 0.92]	0.89 [0.84, 0.93]	0.88 [0.84, 0.93]
CoM position coefficient (β)	1.2 [1.0, 1.3]	1.2 [1.1, 1.3]	1.1 [1.0, 1.3]	Paretic	1.3 [1.2, 1.4]	1.2 [1.1, 1.3]	1.1 [1.0, 1.3]
				Non-paretic	1.2 [1.0, 1.3]	1.1 [0.9, 1.2]	1.0 [0.9, 1.2]
CoM velocity coefficient (β)	0.30 [0.24, 0.37]	0.30 [0.24, 0.37]	0.27 [0.20, 0.34]	Paretic	0.15 [0.10, 0.23]	0.13 [0.07, 0.19]	0.13 [0.07, 0.20]
				Non-paretic	0.24 [0.18, 0.31]	0.21 [0.15, 0.28]	0.22 [0.15, 0.28]
Mediolateral MoS (cm)	1.6 [0.63, 2.6]	2.7 [1.8, 3.7]	3.6 [2.7, 4.6]	Paretic	3.7 [2.7, 4.6]	5.3 [4.4, 6.1]	5.6 [4.8, 6.5]
				Non-paretic	3.4 [2.5, 4.4]	4.4 [3.5, 5.2]	4.6 [3.8, 5.5]
Mediolateral MoS variability (cm)	0.98 [0.38, 1.6]	2.0 [1.3, 2.7]	2.9 [2.1, 3.6]	Paretic	1.5 [0.77, 2.1]	3.5 [2.8, 4.2]	4.6 [3.9, 5.3]
				Non-paretic	1.4 [0.7, 2.0]	3.4 [2.7, 4.1]	4.4 [3.7, 5.2]

Values represent estimated marginal means with 95% confidence intervals. MoS = margin of stability; CoM = centre of mass; xCoM = extrapolated CoM

## Discussion

This study evaluated mediolateral (ML) foot placement control in persons with chronic stroke (PwCS) and controls during walking with continuous ML optic-flow perturbations compared with unperturbed walking. As hypothesized (H1), perturbations increased the variability and average excursion of the xCoM similarly in both groups relative to unperturbed walking, with effects that scaled with perturbation intensity. Following these challenges to ML balance control, PwCS exhibited larger foot placement deviation in both perturbed conditions, consistent with our second hypothesis (H2). However, in contrast to our expectations (H2), no group differences in foot placement deviation were found during unperturbed gait. In line with our third hypothesis (H3) and addressing the primary aim, PwCS showed a larger increase in foot placement deviation than controls at the strong perturbation intensity. Foot placement deviation did not differ significantly between the paretic and non-paretic leg in any condition. Exploratory analyses further showed weak to moderate correlations between foot placement deviation measured during unperturbed and perturbed walking.

### Perturbation effect on CoM dynamics

Consistent with our first hypothesis and previous literature [23, 24, 29–31], the optic-flow perturbations directly affected lateral CoM dynamics in both groups. This was reflected by an increase in step-to-step xCoM variability and larger average xCoM excursions. The magnitude of these changes scaled with perturbation intensity in both groups and across all participants (Fig. 2B), indicating a consistent and amplitude-dependent effect [28]. Furthermore, PwCS exhibited larger overall xCoM excursions, consistent with previous reports of greater ML CoM excursions in this population [21, 43]. Despite this, both groups showed similar increases in xCoM variability and excursion in response to the perturbations. Although visual dependence has been reported to be increased after stroke [26, 44, 45], this comparable increase in CoM excursion and its variability between groups suggests that the perturbations were strong enough to impose a similar challenge to step-to-step balance control in both groups compared to regular walking. Furthermore, participants experienced greater task difficulty with increasing perturbation intensity, with PwCS consistently perceiving the task as more challenging across all conditions. Together, these findings indicate that the optic-flow perturbations elicited a comparable, amplitude-dependent destabilizing effect on ML CoM dynamics in PwCS and controls, providing a valid basis for examining group differences in foot placement control in response to these altered CoM dynamics.

### Foot placement control

Consistent with our second hypothesis (H2), PwCS exhibited a larger foot placement deviation than controls during perturbed walking. However, contrary to our expectations, the between-group difference during unperturbed walking did not reach statistical significance, although a clear trend toward higher foot placement deviation in PwCS was evident. Previous studies have reported impaired CoM-based foot placement control during unperturbed gait after stroke [18, 19]. The absence of a statistically significant baseline group-effect in the present study may reflect the relatively high functional level of our cohort, and/or substantial between-subject variability (Fig. 3A) within the PwCS group, both of which would reduce statistical power to detect smaller baseline differences given the current sample size.

Addressing our primary study aim, and in line with our third hypothesis (H3), PwCS showed a larger increase in foot placement deviation relative to controls when perturbation intensity was high. This indicates that when balance demands increased, PwCS showed larger errors in mediolateral foot placement control in response to perturbation-induced changes in CoM dynamics. Importantly, the regression coefficients ($\:{{\upbeta\:}}_{\mathrm{p}\mathrm{o}\mathrm{s}\:}$and $\:{{\upbeta\:}}_{\mathrm{v}\mathrm{e}\mathrm{l}}\:)$ did not change across conditions in either group. These regression coefficients are often interpreted as foot placement control gains [46], reflecting the strength of the relationship between CoM position or velocity, and the subsequent mediolateral foot placement. This suggests that the perturbations primarily increased step-to-step deviations around the CoM-based foot placement strategy, rather than changing the gains of the strategy. These more pronounced errors in foot placement control observed under destabilizing conditions likely reflect the greater demands imposed on step-to-step balance control during perturbed walking, compared with unperturbed gait. The exploratory analysis illustrates this distinction by revealing weak to moderate associations between unperturbed and perturbed walking, but strong correlations between perturbation intensities, suggesting that foot placement control during perturbed walking is only weakly related to that during unperturbed walking. During unperturbed walking, small CoM deviations are corrected through active foot placement control [12, 47–50]. Although PwCS showed altered foot placement control during steady-state walking in previous work [18–20], such errors do not necessarily result in a loss of balance. PwCS often compensate with wider steps and a larger average MoS, a proactive balance strategy that reduces reliance on reactive balance mechanisms [21, 51, 52]. This proactive strategy was also evident in the present study, with PwCS showing wider steps and a larger average MoS during unperturbed walking compared with controls.

Optic-flow perturbations, however, introduce larger and less predictable CoM deviations than observed during unperturbed walking, which require more rapid, reactive corrections on a step-to-step basis. This shift towards more reactive balance control substantially increases sensorimotor demands, placing greater reliance on reliable sensory integration and rapid motor control [25], both of which are commonly impaired after stroke [15–17, 53]. Consequently, the larger increase in foot placement deviation in PwCS during optic-flow perturbations may reflect a reduced ability to generate timely and accurate foot placement adjustments required for reactive balance control. This may also explain why three PwCS were unable to perform the perturbed conditions without using the handrail. In line with this more impaired step-to-step control, PwCS showed a greater increase in MoS variability during perturbed walking in both legs compared with controls, indicating that impaired foot placement control was accompanied by less consistent lateral stability. Greater MoS variability poses a threat to lateral balance because it increases the likelihood that individual steps become biomechanically unstable, despite a positive average MoS [54]. To counteract this instability risk, both groups increased their step width and average MoS with perturbation intensity, indicating a greater reliance on an already-present proactive, compensatory strategy to maintain balance as conditions became more challenging. Taken together, these findings suggest that PwCS partly rely on a proactive, compensatory balance strategy by widening their base of support and increasing mediolateral MoS to compensate for impairments in step-to-step foot placement control, particularly while walking under destabilizing conditions.

In line with this interpretation, PwCS exhibited a larger average mediolateral MoS compared with controls on the paretic side only during both perturbation conditions, suggesting an increased reliance on this compensatory balance strategy on the side with the least capacity to correct for a potential loss of balance [20, 55]. Contrary to our expectations, however, foot placement control did not differ between the paretic and non-paretic legs. An explanation might be that accurate foot placement control does not only depend on swing-leg control but also on the stance leg, which influences CoM dynamics through push-off and hip abduction [12, 56–58]. Impairments in the paretic stance-leg may therefore indirectly affect the coupling between the CoM and foot placement of the non-paretic swing-leg. Moreover, reduced proprioceptive feedback from the paretic stance-leg may impair CoM state estimation and thereby affect foot placement accuracy of the non-paretic swing-leg. Another explanation is that overall impaired sensory integration in PwCS can further degrade CoM state estimation and add noise to motor commands [25]. This disrupts the feedback-based control that links CoM dynamics to foot placements, potentially reducing control in both legs [59, 60]. Nonetheless, these findings were unexpected considering prior reports of more impaired foot placement control in the paretic leg [19, 20], and highlight the need for experimental studies that disentangle how stance-leg impairments interact with swing-leg foot placement control, particularly under destabilizing conditions.

### Implications

Our results suggest that dynamic balance assessments in PwCS may benefit from including walking conditions that challenge balance control beyond unperturbed, steady-state gait, since stroke-related impairments in step-to-step foot placement control become more pronounced during perturbed gait. Balance control may be challenged through perturbations that directly increase the need for reactive balance responses or by constraining proactive, compensatory strategies such as wider steps to increase the need for step-to-step balance control. The present study demonstrates that manipulating optic-flow can continuously challenge balance control in PwCS across many strides. Although optic-flow perturbations rarely occur in daily life, as opposed to mechanical perturbations such as slips or trips, they provide a safe and controllable method to continuously challenge dynamic balance control across many strides without applying external forces. As such, they offer a task-specific approach to challenge balance control beyond steady-state gait, highlighting the potential of VR-based applications for future experimental studies and rehabilitation after stroke [61]. Adjustable perturbation intensity remains essential, as some PwCS in this study found the predetermined intensities levels too difficult to complete. Finally, our findings support the idea that including perturbation-based elements to dynamic balance assessments may improve the identification of individuals with balance impairments and higher fall risk. This is consistent with previous reports that balance tests including reactive balance components, such as the compensatory stepping items of the Mini-BESTest [62], better distinguish fallers from non-fallers after stroke [63], while noting that these discrete mechanical perturbations differ from the continuous visual perturbations used here and may engage partly different control processes.

### Limitations

This study has several limitations that should be acknowledged. First, individual differences in visual dependence may have contributed to variability in perturbation responses, potentially even differing between groups due to previously reported greater reliance on visual information in PwCS. However, the perturbations elicited similar effects on CoM dynamics in both groups. Furthermore, all participants, including all controls, showed clear effects on CoM dynamics. Therefore, visual dependence is unlikely to have affected the between-group comparisons of foot placement control in response to the perturbations. Second, the three PwCS excluded due to frequent handrail use, as well as the paretic-leg data excluded for one PwCS because foot placement adherence fell below the pre-defined threshold (R² < 0.5), represent non-random exclusions of individuals who were likely more severely affected, more challenged by the perturbations, and/or more fearful of falling. Importantly, their exclusion is unlikely to explain the observed group-effects, as including them would likely have increased, rather than reduced, the between-group differences. Third, we included relatively high-functioning PwCS in the analysis who were able to walk independently (FAC ≥ 3) and complete perturbation trials without using the handrail. The findings should not be generalized to lower-functioning individuals. Finally, we note that our results focus on a single balance control mechanism. Other balance control mechanisms, such as ankle-based modulation during stance [64], likely also contributed to the observed balance responses but were not quantified in the present study.

## Conclusion

PwCS exhibited impaired mediolateral foot placement control during walking, which worsened as reactive balance demands increased during continuous optic-flow perturbations. Together with the weak association between foot placement control under unperturbed and perturbed conditions, these findings indicate that challenging balance control amplifies stroke-related impairments in step-to-step balance control compared to regular, steady-state walking. Optic-flow perturbations can challenge balance across many strides, supporting the potential of VR-based applications in post-stroke rehabilitation.

## Supplementary Information

Below is the link to the electronic supplementary material.

Supplementary Material 1.

## Data Availability

The dataset collected and analyzed during the current study is available from the corresponding author on reasonable request.

## Abbreviations

CoM: Centre of mass
HC: Healthy controls
ML: Mediolateral
PwCS: Person(s) with chronic stroke
RMSE: Root mean square error
VR: Virtual reality
xCoM: Extrapolated centre of mass
