# Supplementary material for: Foot placement control impairments in persons with chronic stroke become more evident during optic-flow perturbations
Source: J Neuroeng Rehabil. 2026 Apr 3;23:160. doi: 10.1186/s12984-026-01967-y (PMC13173955; doi:10.1186/s12984-026-01967-y)
Supplement: Supplementary file 1 — Supplementary Material 1. [file 12984_2026_1967_MOESM1_ESM.docx]

**Supplemental Table S1.** Estimated marginal means and 95% confidence intervals of spatiotemporal gait parameters in controls and persons with chronic stroke (PwCS) in unperturbed walking and during continuous optic-flow perturbations with moderate or strong intensity.

|  | Controls (n=16) | | | Persons with chronic stroke (n=17) | | | |
| --- | --- | --- | --- | --- | --- | --- | --- |
|  | **Unperturbed** | **Moderate perturbation intensity** | **Strong perturbation intensity** |  | **Unperturbed** | **Moderate perturbation intensity** | **Strong perturbation intensity** |
| *Step frequency (steps/s)* | 1.3 [1.2, 1.4] | 1.5 [1.4, 1.6] | 1.6 [1.5, 1.7] |  | 1.6 [1.5, 1.6] | 1.7 [1.6, 1.8] | 1.8 [1.7, 1.8] |
| *Step width (cm)* | 10.1 [7.6, 12.7] | 12.1 [9.6, 14.7] | 14.4 [11.9, 17.0] |  | 15.4 [12.9, 17.8] | 18.0 [15.5, 20.5] | 18.3 [15.8, 20.8] |
| *Step width variability (cm)* | 1.0 [0.6, 1.4] | 1.8 [1.5, 2.2] | 2.88 [2.5, 3.3] |  | 1.9 [1.5, 2.2] | 2.8 [2.4, 3.1] | 3.2 [2.9, 3.7] |
| *Step length (cm)* | 47.5 [44.5, 50.4] | 42.7 [39.8, 45.7] | 39.5 [36.6, 42.5] | **Paretic** | 46.9 [44.0, 49.8] | 42.4 [39.5, 45.3] | 41.8 [38.8, 44.7] |
|  |  |  |  | **Non-paretic** | 44.1 [41.3, 47.0] | 40.4 [37.5, 43.3] | 38.8 [35.9, 41.8] |
| *Step length variability (cm)* | 2.6 [1.9, 3.3] | 3.8 [3.1, 4.4] | 5.1 [4.4, 5.8] | **Paretic** | 3.2 [2.5, 3.9] | 5.3 [4.6, 6.0] | 6.3 [5.6, 7.0] |
|  |  |  |  | **Non-paretic** | 3.3 [2.7, 4.0] | 5.1 [4.5, 5.8] | 6.1 [5.4, 6.8] |
| *Stance phase duration (s)* | 1.1 [1.0, 1.2] | 0.98 [0.92, 1.0] | 0.91 [0.85, 0.97] | **Paretic** | 0.92 [0.86, 1.0] | 0.84 [0.78, 0.89] | 0.82 [0.76, 0.87] |
|  |  |  |  | **Non-paretic** | 0.96 [0.90, 1.0] | 0.88 [0.82, 0.93] | 0.85 [0.80, 0.91] |
| *Swing phase duration (s)* | 0.44 [0.42, 0.47] | 0.40 [0.38, 0.43] | 0.38 [0.36, 0.40] | **Paretic** | 0.40 [0.37, 0.42] | 0.37 [0.35, 0.40] | 0.37 [0.34, 0.39] |
|  |  |  |  | **Non-paretic** | 0.36 [0.34, 0.38] | 0.33 [0.31, 0.36] | 0.33 [0.30, 0.35] |
